# Persistence of *TEL-AML1 *fusion gene as minimal residual disease has no additive prognostic value in CD 10 positive B-acute lymphoblastic leukemia: a FISH study

**DOI:** 10.1186/1756-8722-1-17

**Published:** 2008-10-17

**Authors:** Eman Mosad, Hosny B Hamed, Rania M Bakry, Azza M Ezz-Eldin, Nesrine M Khalifa

**Affiliations:** 1 Clinical Pathology Department, South Egypt Cancer Institute, Assiut University, Assiut, Egypt; 2 Clinical Pathology Department, Assiut University Hospital, Assiut University, Assiut, Egypt; 3 Pediatric Oncology Department, South Egypt Cancer Institute, Assiut University, Assiut, Egypt

## Abstract

**Objectives:**

We have analyzed t(12;21)(p13:q22) in an attempt to evaluate the frequency and prognostic significance of *TEL-AML1 *fusion gene in patients with childhood CD 10 positive B-ALL by fluorescence in situ hybridization (FISH). Also, we have monitored the prognostic value of this gene as a minimal residual disease (MRD).

**Methods:**

All bone marrow samples of eighty patients diagnosed as CD 10 positive B-ALL in South Egypt Cancer Institute were evaluated by fluorescence in situ hybridization (FISH) for t(12;21) in newly diagnosed cases and after morphological complete remission as a minimal residual disease (MRD). We determined the prognostic significance of *TEL-AML1 *fusion represented by disease course and survival.

**Results:**

*TEL-AML1 *fusion gene was positive in (37.5%) in newly diagnosed patients. There was a significant correlation between *TEL-AML1 *fusion gene both at diagnosis (r = 0.5, P = 0.003) and as a MRD (r = 0.4, P = 0.01) with favorable course. Kaplan-Meier curve for the presence of *TEL-AML1 *fusion at the diagnosis was associated with a better probability of overall survival (OS); mean survival time was 47 ± 1 month, in contrast to 28 ± 5 month in its absence (P = 0.006). Also, the persistence at *TEL-AML1 *fusion as a MRD was not significantly associated with a better probability of OS; the mean survival time was 42 ± 2 months in the presence of MRD and it was 40 ± 1 months in its absence. So, persistence of *TEL-AML1 *fusion as a MRD had no additive prognostic value over its measurement at diagnosis in terms of predicting the probability of OS.

**Conclusion:**

For most patients, the presence of *TEL-AML1 *fusion gene at diagnosis suggests a favorable prognosis. The present study suggests that persistence of *TEL-AML1 *fusion as MRD has no additive prognostic value.

## Background

Acute lymphoblastic leukemia (ALL) is the most common malignancy of childhood. Cure of many of these children is difficult to predict and is considered an individual response of the patient to chemotherapy. It is likely that this clinical heterogeneity reflects a diverse pathogenesis of leukemia. The molecular basis of childhood ALL is largely unknown. Furthermore, it is likely that significant advance in the treatment of childhood ALL will be dependent on a better understanding of the molecular events that cause the disease [1,2].

A recurrent t(12;21)(p13:q22) has been described in several human ALLs. In this translocation the *TEL *gene fuses to *AML1*; a gene previously cloned from translocation breakpoints in acute myeloid leukemia. These abnormalities consist of both translocations and deletions. The frequency of t(12;21) was estimated as to be 15–35% in childhood ALL. This translocation has been recognized as the most common chromosomal aberration in childhood ALL [2-4]. All (95–100%) of *TEL-AML1 *positive ALL patients found to has a consistent cell surface immunophenotype. (B lineage ALL based on the expression of HLA-DR, CD 10 and CD 19) [2,4]. Thus, we raised a question if the opposite is true meaning that if CD 10 positive B-ALL immunophenotype will have a similarly high incidence of positive *TEL-AML1 *fusion gene?. Accordingly can we use this fusion gene as a minimal residual disease (MRD) in this specific subgroup of B-ALL.

It was also reported that patients with the *TEL-AML1 *fusion have a high sensitivity to chemotherapy [4-6]. Other investigators have reported that almost 10–28% of relapsed pediatric ALL patients express the *TEL-AML1 *fusion, but the relapse of patients with the *TEL-AML1 *fusion is not always associated with a poor prognosis [7-9]. However, some patients with the *TEL-AML1 *transcripts and additional molecular lesions had poor outcomes [10]. So, the prognostic significance of *TEL-AML1 *transcript remains controversial.

Patients with a poor treatment response by morphologic criteria have a high risk of relapse [11,12]. But morphologic studies will only identify a minority of those children with ALL who eventually fail. Minimal residual disease (MRD) has been of prognostic value in children with ALL. Several studies have shown that children with a high leukemic cell burden at the end of induction therapy have an inferior outcome compared to children with a lower leukemic cell burden [13-18]. The investigation of MRD using *TEL-AML1 *fusion gene as a marker has been carried out on a limited number of patients to date although it is a minor examination. The relation between relapse and the persistence of detectable MRD show heterogeneity [19,20]. As this translocation is often difficult to detect by conventional G-banding analysis, in addition many patients with ALL were diagnosed as normal karyotype or could not examined for karyotype by classic cytogenetic analysis. In particular fluorescence in situ hybridization (FISH) analysis has been applied to hematopoietic malignancies with subtle or complex chromosomal aberrations which are difficult or impossible to detect by standard cytogenetic analysis [3]. Therefore, we conducted a retrospective study to determine the frequency and prognostic significance of *TEL-AML1 *fusion in CD 10 positive B-ALL, and to clarify whether the persistence of the *TEL-AML1 *fusion gene as a MRD has an additive value.

## Methods

### Patients and Samples

Bone marrow (BM) samples were obtained from 80 CD 10 positive B-ALL patients aged from 3 to 11 years; mean age was 7.4 ± 2, diagnosed at our Institute between 2002 and 2004 and followed up till 2006. Diagnosis was performed according to the standard procedures; French American British (FAB) classification of lymphoblastic leukemia and determination of immunophenotypic markers. They were B precursor ALL patients diagnosed as common and preB-ALL by flowcytomety (expressing CD19, CD 10 and HLA-DR). Patients were considered in the standard risk category if they were aged 1–9 years, had white blood cell count < 50,000 per micro liter, or had central nervous system affection. The remaining patients were considered as high risk. Patients were treated according to modified Berlin-Frankfurt-Munster (BFM-90) ALL protocol.21 t(12;21) was evaluated by FISH in newly diagnosed cases (80 patients) and after morphological remission in patients who were positive for t(12;21) as a MRD (30 patients) and we determined the prognostic significance of *TEL-AML1 *fusion represented by disease course and survival and we clarified if the persistence of the *TEL-AML1 *fusion gene as MRD had an additive prognostic value. Five normal BM samples were taken as a control and the level of *TEL-AML1 *fusion by FISH estimated as 1 ± 0.2%. Therefore, the cut-off level used in this study was 1.2%. The study was approved by our faculty ethical committee and was adherent to the regulations of the declaration of Helsinski.

### Response Criteria

Complete remission (CR) was defined as the complete disappearance of all tumor masses confirmed at clinical examination, or X-rays, and ultrasound studies; a normal BM examination and pathology; and no evidence of CNS disease by cerebrospinal fluid analysis.

The disease course was assessed by ranking patients according to their response to treatment into 4 categories; CR1, CR2, CR3, and resistance and/or death. CR1 patients were those who achieved first complete remission. Patients who received therapy for their first or second relapse and achieved <5% blasts in the marrow and had extramedullary sites of leukemia were considered to be in second or third remission (CR2 or CR3). Patients whose marrow showed >5% blasts with or without evidence of extramedullary disease were considered to be in relapse.

### Detection Of t(12;21) By FISH Analysis In ALL Patients

In situ hybridization (ISH) is a technique that allows the visualization of a specific nucleic acid sequences within a cellular preparation. Specifically DNA FISH involves the precise annealing of a single standard fluorescently labeled DNA probe to complementary target sequences. The hybridization of the probe with the cellular DNA site is visible by direct detection using fluorescence microscopy.

After 24 hours of unstimulated culture, samples were fixed. Interphase cells were attached to glass slides using standard cytogenetic protocol. The resulting specimen DNA was denaturated to its single strand form and then allowed to hybridize with LSI *TEL/AML1 *ES Dual Color probe to detect t (12;21) 12p13 spectrum green/21q22 spectrum orange catalog 32-191005-Vysis. Following hybridization, the excess and unbound probe was removed by a series of washes and the chromosomes and nuclei were counter stained with DNA specific stain DAPI (4.6 diamidino-2-phenylindole) that fluoresces blue. The expected pattern in normal nucleus hybridized with *TEL/AML1 *probe is two orange, two green (2O2G). In the nucleus harboring the t(12;21), the probe hybridized to a nucleus containing the t (12;21) showing one green (native *TEL*), one large orange (native *AML1*), one smaller orange (ES) and one fused orange/green (20IGIF) signal pattern. The Microscopy and photography were conducted using a Zeiss Axiovert 200 fluorescence microscope fitted with a high resolution Leica CCD camera. Images were processed using Leica CW4000 imaging system and software (Leica, Germany).

### Statistical Methods

The study cutoff time limit was September 2006. Overall survival (OS) was calculated from the first day of chemotherapy to the date of last follow up contact for patients who were alive. All data were analyzed using SPSS (Statistical Program for Social Sciences version 11 for windows, 2001, SPSS Inc., Chicago, IL, USA). Correlations are done using Pearson correlation test. Categorical variables were compared using chi-square test with Fisher's Exact correction. OS is estimated with the Kaplan-Meier method. A *P *value < 0.05 was considered to be significant.

## Results

Eighty ALL patients were enrolled in the study at our Institute between 2002 and 2006. They males were (n = 56; 70%) and females were (n = 24; 30%), mean age 7.4 ± 2 years they were 44 patients L1 (55%) and 36 L2 (45%). They were all B-lineage ALL positive for CD 10 and CD 19 by immunophenotyping (common and pre B-ALL). Most of our patients were in the standard risk (n = 64; 80%), while (n = 16; 20%) were in the high risk category. The karyotypes: Seven metaphases were available for cytogenetic analysis and they were normal. A precise karyotype was not obtained from other patients because of poor morphology of metaphases.

**Figure 1:**
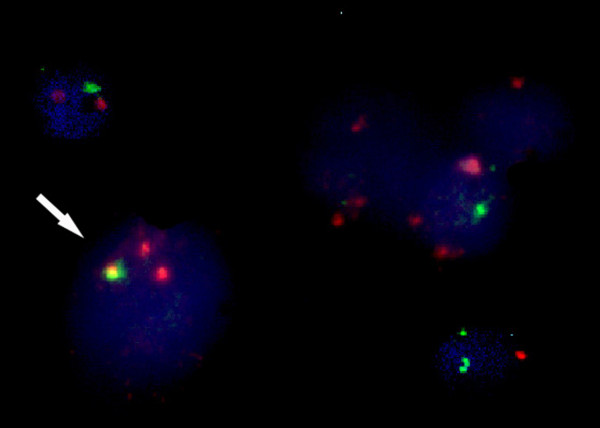
**TEL-AML-I fusion gene by FISH**. It shows a fused yellow signal on the der (21) chromosome in the interphase nuclei of leukemic cells.

*TEL-AML1 *fusion gene was evaluated by FISH which showed a fused yellow signal (Figure 1) on the der (21) chromosome in the metaphase and on the interphase nuclei of leukemic cells. It was measured in newly diagnosed cases (Table 1) and it was positive in 30/80 (37.5%) determining its frequency in B-lineage ALL positive for CD 10 and CD 19. The mean percent of *TEL-AML1 *fusion gene was 50 ± 22% estimated in 300 interphase cells. A control was performed using, five normal bone marrow samples and the cut-off level in this method was estimated to be 1.2%. There was a favorable significant correlation between *TEL-AML1 *fusion gene and disease course (*r *= 0.5, *P *= 0.003). Of particular interest was the observation that 10/50 (20%) of patients lacking the *TEL-AML1 *fusion had a very bad course (eight children did not achieve a complete remission after induction chemotherapy (resistant) and two achieved CNS relapse and died). No significant correlation was detected between the presence of *TEL-AML1 *fusion gene at diagnosis and peripheral WBC count, age, sex, organs, FAB classification, central nervous system disease, and risk category. We analyzed the patients who were positive for the presence of *TEL-AML1 *fusion at diagnosis (n = 30) to detect its persistence as a MRD in patients who entered in complete remission morphologically (Table 2). It was positive in (n = 15/30; 50%) patients. The mean percent of *TEL-AML1 *fusion gene was 7 ± 2% estimated in 300 interphase cells. The persistence of *TEL-AML1 *fusion gene as a MRD, was correlated with a favorable course (*r *= 0.4, *P *= 0.01). To be noticed that (n = 12/15; 80%) of MRD positivity were in CRI.

**Table 1:** Interpahse FISH results of the patients with the TEL-AML1 fusion gene at diagnosis

		**Clinical course**					
		
		CR1	CR2	CR2, CNS relapse and death	Resistant and death	**Total**	***P***
**T (12; 21) at Diagnosis**	Yes	20	9	1	0	30	0.003
	
	No	9	31	2	8	50	

**Total**		**29**	**40**	**3**	**8**	**80**	

FISH, fluorescence in situ hybridization; CR1, first complete remission; CR2, second complete remission; CNS, involvement of the central nervous system.

**Table 2:** Interpahse FISH results of the patients with the TEL-AML1 fusion after complete remission as MRD

		**Clinical course**					
		
		CR1	CR2	CR2, CNS relapse and death	Resistant and death	**Total**	***P***
**t (12; 21) at Remission (MRD)**	Yes	12	2	1	-	15	0.01
	
	No	6	9	-	-	15	
	
**Total**		**18**	**11**	**1**	-	**30**	

FISH was performed to the patients with a positive t(12;21) at the time of diagnosis who passed to remission; a total number of 30 patients. FISH, fluorescence in situ hybridization; CR1, first complete remission; CR2, second complete remission; CNS, involvement of the central nervous system

**Figure 2:**
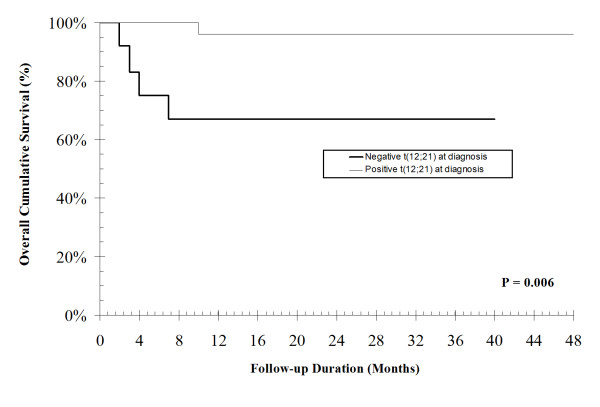
**TEL-AML1 fusion at diagnosis**. Kaplan-Meier curve for the presence of TEL-AML1 fusion at diagnosis as a predictor of overall cumulative survival.

**Figure 3:**
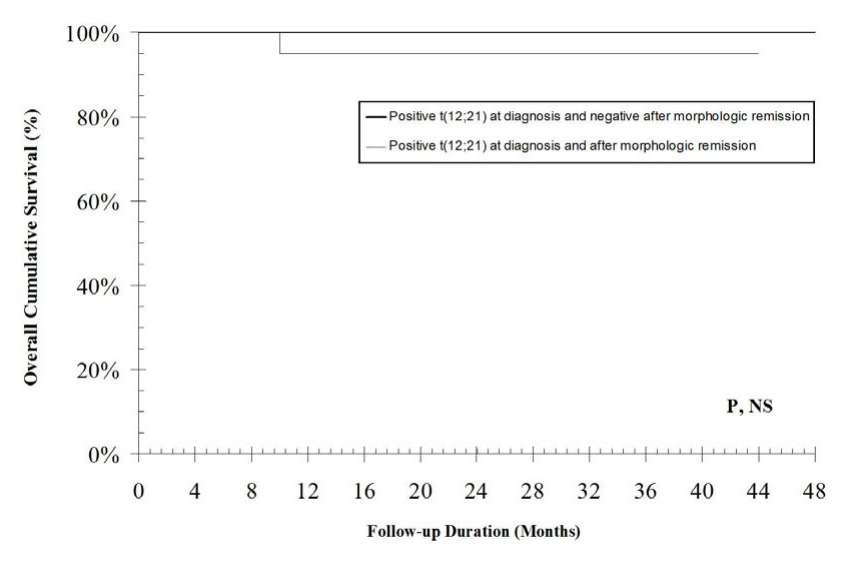
**TEL-AML1 fusion as a minimal residual disease**. Kaplan-Meier curve for the persistence of TEL-AML1 fusion as a minimal residual disease (MRD) as a predictor of overall cumulative survival.

Kaplan-Meier curve for the presence of *TEL-AML1 *fusion at the diagnosis was associated with a better probability of OS (Figure 2); mean survival time was 47 ± 1 month, in contrast to 28 ± 5 month in its absence (*P *= 0.006). Also, the persistence at *TEL-AML1 *fusion as a MRD was not significantly associated with a better probability of OS (Figure 3); the mean survival time was 42 ± 2 months in the presence of MRD and it was 40 ± 1 months in its absence. So, persistence of *TEL-AML1 *fusion as a MRD had no additive prognostic value over its measurement at diagnosis in terms of predicting the probability of OS.

## Discussion

The *TEL *gene encodes a member of the ETS family of transcription factors and is rearranged in a wide variety of hematological malignancies. In particular, *TEL *is fused to the platelet-derived growth factor receptor β in CMML, to the ABL tyrosine kinase in acute myeloid leukemia and ALL, and to the product of the MNI gene in myeloproliferative disorders. *AML-I *is the DNA-binding subunit of the transcription factor complex core binding factor (CBF-β). It is frequently rearranged in myeloid malignancy either through fusion to ETO as a result of t(8;21)(q22:q22) or to EVII, MDS1, or EAP as a result of t(3;21)(q26:q22).[2,22] The frequent involvement of *TEL *and *AML-I *in chromosomal translocations suggests that these genes play important roles in the pathogenesis of human leukemia. In t(12;21) a high level of expression of the hybrid protein that contains the functional domains of *AML-I *under the transcriptional control of the *TEL *promoter may be involved in oncogenic transformation [2,22]. In this study we demonstrated that the frequency of *TEL-AML1 *fusion in B-Lineage CD10 positive ALL was 37.5%. versus 30% in a previous study included multicentres and larger number of patients.23 In our data (22%)of patients with t(12;21) were CD34-positive, indicating that the leukemic cells originated from primitive hematopoietic cell similar to those of ALL patients with t(9;22) or 11q23 abnormalities [2]. We also, found that 67% of the t(12;21) positive patients were in (CR1), indicating a favorable course, as previously reported [1,2].

The relationship between the *TEL-AML1 *fusion and a favorable prognosis represented by survival has already been described [2,9]. Rubnitz et al [9] reported that the survival at five years follow-up of a group with the *TEL-AML1 *fusion was 91 ± 5%. These patients with positive *TEL-AML1 *fusion who achieved a favorable prognosis were found to be younger, without hyperleukocyosis, with the CD 10 positive B precursor ALL immunophenotyping and chemosensitive [2]. Also, a recent report studying the prognosis of relapsed patients showed an outcome consistent with ours. The median duration of remission of relapsed TEL-AML1-positive patients was reported to be 42.5 versus 27 months in those lacking the gene; P = 0.0001 [24]. Our study was consistent with the pervious studies as *TEL-AML1 *fusion at the diagnosis was associated with a better probability of overall survival [2,8,9]. On the other hand, other studies have reported that some patients with *TEL-AML1 *transcript had a poor outcome [25]. In many cases, *TEL-AML1 *transcripts detected by RT-PCR and Southern blotting in childhood ALL disappeared soon after the start of chemotherapy [6,26]. Others reported that a patient with additional molecular lesions with p16 homozygous deletion in addition to *TEL-AML1 *transcript relapsed usually late, and the survival was ultimately favorable [8,9]. An analysis of late or off-treatment relapse of *TEL-AML1 *positive ALL suggested that leukemic cells in relapse were not derived from the dominant clone at diagnosis. It represents a transformation of cells belonging to a persistent preleukemic clone that was generated by *TEL-AML1 *fusion in utero and survived chemotherapy [27].

The progress in treatment of ALL patients without conventional risk factors has been hampered by the inability to predict relapse after patients achieved a complete remission [19]. Whereas in large prospective studies on childhood ALL, residual disease is a powerful indicator of treatment outcome [20,28]. In this study, MRD was detectable in 50% of patients of CD 10 positive B-ALL after 4 to 6 weeks of induction therapy. Whereas, in a prospective study on childhood ALL, MRD was detectable in 25% to 58% of patients after the same period of induction therapy [20]. It was reported that the frequency of MRD positivity is high after induction and decreases gradually during consolidation and maintenance phase being in some genes 88% during early induction to 13% at week 52 [19]. If MRD as a marker was detected, the general opinion is that it could become a risk factor for relapse [20,28]. In contrast, MRD lasts among some patients in long-term remission in other forms of childhood acute leukemia like t(15;17) and t(8;21). [32,33] Cayuela et al [26], reported that one out of seven patients with the *TEL-AML1 *transcript, serially evaluated, exhibited persistence of detectable MRD over eight months, and that all the patients were in continuous complete remission. This study was consistent with that reported by others [26,29,30] that several patients were found to be positive for *TEL-AML1 *fusion, but the persistence of detectable MRD was not associated with a better probability of OS. Therefore, the relationship of the MRD level of *TEL-AML1 *fusion and prognosis shows heterogeneity and further investigation is required to evaluate their association and to design risk adapted therapeutic approaches.

## Conclusion

*TEL-AML1 *fusion gene detected by FISH in newly diagnosed cases of CD 10 positive B-ALL is considered a favorable prognostic marker with a better course. The persistence of *TEL-AML1 *fusion gene as a MRD has no additive prognostic value. Considering the cost-benefit ratio *TEL-AML1 *fusion gene done once at diagnosis gives sufficient prognostic information. However, much research about the biologic and clinical significance of *TEL-AML1 *as MRD in CD 10 positive ALL is needed to determine how to best integrate *TEL-AML1 *testing into routine patient care.

## Abbreviations

ALL: Acute lymphoblastic leukemia; CD: cluster differentiation; CMML: chronic myelomonocytic leukemia; CR: Complete remission; FISH: fluorescence in situ hybridization; HLA: human leucocytic antigen; MRD: minimal residual disease; OS: overall survival; T: translocation.

## Competing interests

The authors declare that they have no competing interests.

## Authors' contributions

EM participated in study design, conducted FISH technique, statistical analysis and wrote the manuscript. HB, RMB, AME-E participated in study design, in conducting FISH technique, critical manuscript revision. NMK participated in study concept and was responsible for the clinical aspect of the work as regards patients' clinical assessment, management and follow-up. All authors read and approved the manuscript.
